# Sexual Cannibalism: High Incidence in a Natural Population with Benefits to Females

**DOI:** 10.1371/journal.pone.0003484

**Published:** 2008-10-22

**Authors:** Rubén Rabaneda-Bueno, Miguel Á. Rodríguez-Gironés, Sara Aguado-de-la-Paz, Carmen Fernández-Montraveta, Eva De Mas, David H. Wise, Jordi Moya-Laraño

**Affiliations:** 1 Dpto. de Ecología Funcional y Evolutiva, Estación Experimental de Zonas Áridas (Consejo Superior de Investigaciones Científicas), Almería, Spain; 2 Dpto. de Psicología Biológica y de la Salud, Universidad Autónoma de Madrid, Cantoblanco, Madrid, Spain; 3 Department of Biological Sciences and Institute for Environmental Science and Policy, University of Illinois at Chicago, SES (M/C 066), Chicago, Illinois, United States of America; The University of New South Wales, Australia

## Abstract

**Background:**

Sexual cannibalism may be a form of extreme sexual conflict in which females benefit more from feeding on males than mating with them, and males avoid aggressive, cannibalistic females in order to increase net fitness. A thorough understanding of the adaptive significance of sexual cannibalism is hindered by our ignorance of its prevalence in nature. Furthermore, there are serious doubts about the food value of males, probably because most studies that attempt to document benefits of sexual cannibalism to the female have been conducted in the laboratory with non-natural alternative prey. Thus, to understand more fully the ecology and evolution of sexual cannibalism, field experiments are needed to document the prevalence of sexual cannibalism and its benefits to females.

**Methodology/Principal Findings:**

We conducted field experiments with the Mediterranean tarantula (*Lycosa tarantula*), a burrowing wolf spider, to address these issues. At natural rates of encounter with males, approximately a third of *L. tarantula* females cannibalized the male. The rate of sexual cannibalism increased with male availability, and females were more likely to kill and consume an approaching male if they had previously mated with another male. We show that females benefit from feeding on a male by breeding earlier, producing 30% more offspring per egg sac, and producing progeny of higher body condition. Offspring of sexually cannibalistic females dispersed earlier and were larger later in the season than spiderlings of non-cannibalistic females.

**Conclusions/Significance:**

In nature a substantial fraction of female *L. tarantula* kill and consume approaching males instead of mating with them. This behaviour is more likely to occur if the female has mated previously. Cannibalistic females have higher rates of reproduction, and produce higher-quality offspring, than non-cannibalistic females. Our findings further suggest that female *L. tarantula* are nutrient-limited in nature and that males are high-quality prey. The results of these field experiments support the hypothesis that sexual cannibalism is adaptive to females.

## Introduction

Sexual cannibalism, a behaviour in which one member of a courting or copulating male-female pair consumes the other, may be widespread among some arthropods [1]. A female can kill a male either before, during, or after mating [1], [2]. Pre-mating sexual cannibalism entails extreme sexual conflict: females may accrue nutritional benefits by killing and consuming approaching males instead of mating with them [3], whereas males, who must approach females in order to copulate, risk being killed by the females they approach [4]. Pre-mating sexual cannibalism may also be a form of mate choice, in which females kill and consume males that they estimate to be of low quality as sires [1], [5]. This paper deals with the foraging benefits of pre-mating sexual cannibalism [3].

The adaptive value of pre-mating sexual cannibalism is controversial largely because there is little supporting evidence that killing and eating a potential mate benefits females at all [3], [6]–[11]. Fuelling the controversy is the fact that most studies seeking to determine the adaptive value of sexual cannibalism to females are not conducted under natural conditions [6], [8]–[12] and there are only few correlational studies in the field [13], [14]. Knowing the rate at which sexual cannibalism occurs in nature is crucial to understanding its ecological and evolutionary significance; however, the rate of sexual cannibalism in nature is largely unknown [1], [15]. High rates of sexual cannibalism in staged encounters in the laboratory may be a laboratory artefact. Jackson (1980) proposed that the actual rate of sexual cannibalism among spiders in nature may not be as high as previously thought [16]. For instance, if females in the laboratory lack some essential nutrients, or if males in a cage cannot escape the female's attack, observed rates of cannibalism may be artificially high. When sexual cannibalism is pre-copulatory, natural selection should favour males that avoid cannibalistic females, leading to adaptive male behaviors such as approaching females at times of the day or season when they are less aggressive [17], [18], or being able to recognize, and preferentially approach, the less aggressive females in the population [13]. The circumstances of staged encounters in the laboratory often do not permit the expression of such adaptive male behaviours.

Laboratory experiments on sexual cannibalism have never been conducted with a spectrum of natural prey as alternative food sources. The quality and amount of prey in a laboratory experiment may be very different from that in the wild. This is not a trivial issue, because increasing numbers of studies reveal that differences in prey quality may affect the survival, growth and fecundity of predatory arthropods [19]–[22], and that predatory arthropods may feed differentially according to nutrient needs [23], [24]. Thus, the quality of alternative prey in a female's environment may influence her proclivity towards sexual cannibalism.

Therefore, in order to understand more thoroughly both the ecological implications of sexual cannibalism and its evolutionary origins and evolutionary maintenance, i.e. whether or not it is likely to be an evolved, adaptive behaviour, field experiments are needed to document that 1) pre-mating sexual cannibalism occurs at substantial rates in nature, and 2) females that are exposed to the spectrum of alternative prey present in their natural environment benefit from sexual cannibalism.

The Mediterranean tarantula (*Lycosa tarantula*) is a territorial and cannibalistic burrowing wolf spider [25] that is well suited for experiments on pre-mating sexual cannibalism. First, field experiments have established that female *L. tarantula* are food-limited in nature [14], [26]. Second, observations in nature and field experiments reveal that female *L. tarantula* can be sexual cannibals [13], [25]. Third, indirect evidence from a field experiment [14] suggests that females appear to compensate for the effects of food limitation experienced as juveniles by cannibalizing males, although it remains to be established conclusively that the apparent compensation for food limitation is actually due to sexual cannibalism. Fourth, sexual size dimorphism in *L. tarantula* is relatively small [27], [28], which makes males a potentially good meal for females [14], especially in comparison with natural prey, which are on average several times smaller [25]. And lastly, females can easily be induced to take up residence in standardized burrows constructed at pre-determined locations in the field, which greatly facilitates density manipulations and behavioural observations [25].

Here we present the results of three field experiments and a supporting laboratory experiment with *L. tarantula* designed to reveal whether or not pre-mating sexual cannibalism (1) occurs in nature at natural rates of encounter between males and females; (2) is relatively frequent, or instead, is a rare occurrence; (3) is more frequent when males are more abundant; and (4) increases female fitness by enhancing fecundity and/or offspring quality. In the first field experiment we manipulated male availability by establishing three different densities of males in large plots in which females at natural density were exposed to natural densities of alternative (i.e. non- *L. tarantula*) prey. In this experiment we measured both the rate of sexual cannibalism and the frequency of mating. In a second field experiment, individual females in enclosures approximating female territory size [29], [30] were provided with natural prey at rates mimicking rates of supply in nature, with half of these females also being permitted to feed on a cannibalized male. A third field experiment, and a laboratory experiment, compared the performance of the progeny of females that had fed on a male in addition to natural prey, to that of the offspring of females that had consumed natural prey but no male *L. tarantula*. These experiments revealed that sexual cannibalism by female *L. tarantula* occurs frequently in nature, and that females that have fed on natural heterospecific prey still obtain clear fitness benefits from also including a single male *L. tarantula* in their diet.

## Results

### Field Experiment 1: Natural rates of pre-copulatory sexual cannibalism and the influence of male availability on cannibalism rate

In large field plots that enclosed female *L. tarantula* in burrows at natural densities and that spanned three different male-density treatments, a third of the females (24/72) cannibalized at least one male; five females cannibalized two males. Cannibalism was almost entirely pre-copulatory (28/29 cannibalistic events).

**Figure 1:**
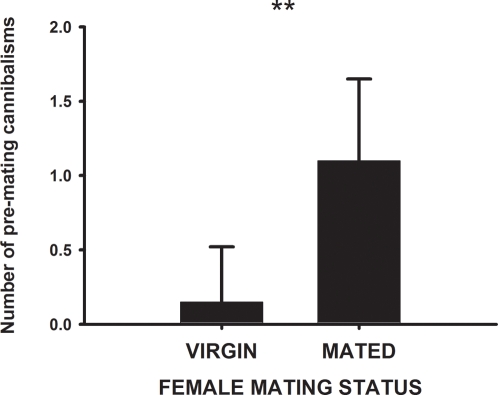
Female mating status affects the rate of pre-copulatory sexual cannibalism in *L. tarantula*. Rates of pre-mating sexual cannibalism (number of cannibalistic events per female) for 20 females that cannibalized at least one male. We compare the mean number of cannibalistic events for the same females as virgins and after they had mated once. Bars and error bars represent mean and standard errors, respectively. **, *P*<0.01. Refer to the text for more details of the statistical analysis.

Females did not tend to attack males indiscriminately–mated females were more likely than virgin females to kill a potential mate. Among those females who killed a potential mate before mating and who also mated at least once during the experiment (20 out of 24 cannibalistic females), mated females showed a 3.4× higher rate of pre-mating sexual cannibalism than virgins (Wilcoxon matched-pairs test, Z = 3.2, *P* = 0.0015). Only 15% (3/20) of this group of cannibalistic females killed a male before their first mating (Fig. 1). This result is not likely due to a difference in how many males were available for virgin and mated females, as the numbers of males that were released in the plots before (10.9±1.6) and after (14.4±1.6) each female mated for the first time were only 1.4× higher after mating and not significantly different (Wilcoxon matched-pairs test, *Z* = 1.1, *P* = 0.29). In addition, the number of males released before or after mating was not significantly associated with the number of cannibalistic events before or after mating (both Spearman correlations *r*_s_<0.25; *P*>0.27). We also tested whether there were significant effects of plot or the male-availability treatment on whether or not mating status affected the probability that a female was cannibalistic, and found no significant effects (both *P*'s in the model>0.45). In the above analyses, 3 cannibalistic females could not be included because they killed the male while they were virgin and subsequently never mated. Thus, the number of virgin females that killed a male is 8% of all virgins (6 out of 72, the total number of virgin females introduced into the plots), and the number of females who first killed a male when they were virgins is 25% of all cannibalistic females (6/24).

**Figure 2:**
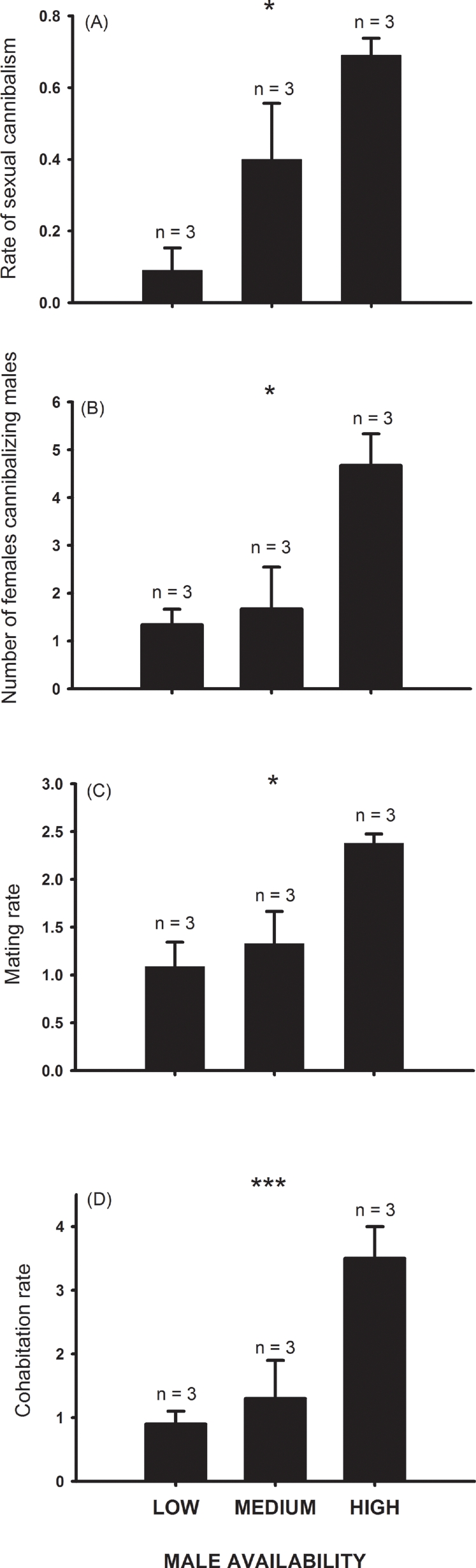
The effect of male availability on rates of sexual cannibalism, mating and cohabitation in female *Lycosa tarantula*. Solid bars and error bars represent means (calculated as the mean of the plot-level values of the response variables) and standard errors for the three male-density treatments, respectively. (A) Rate of sexual cannibalism–average number of cannibalistic events observed among females and averaged among plots (*F*_2, 6_ = 8.7, *P* = 0.017); (B) Number of cannibalistic females per plot (*F*_2, 6_ = 7.6, *P* = 0.02); (C) Mating rate–average number of copulations per plot (*F*_2, 6_ = 7.6, *P* = 0.02); and (D) Cohabitation rate–average number of males observed within 20 cm of a female burrow within each plot (*F*_2, 6_ = 25.8, *P*<0.001). Symbols denote significant differences among groups: *, *P*<0.05; *** *P*<0.001. More powerful tests for linear trends can be found in the text.

Our manipulation of male densities revealed that as the number of males per female in the population increased, the rate of sexual cannibalism and the number of matings per female also increased (Fig. 2A, C). Differences among treatment groups are significant for all response variables (*P*'s<0.05, GLM). For simplicity, however, we present results only for the linear trends, i.e. the expected increase in a response variable with increasing male availability. The rate of cannibalism (mean number of cannibalistic events per plot) increased linearly from the low to high male-density treatments (orthogonal linear contrast, *F*_1, 6_ = 17.4, *P* = 0.006, Fig. 2A), as did the number of cannibalistic females per plot (orthogonal linear contrast, *F*_1, 6_ = 30.0, *P* = 0.002, Fig. 2B). The mean number of copulations per plot also displayed a linear trend (orthogonal linear contrast, *F*_1, 6_ = 13.5, *P* = 0.01; Fig. 2C). The rate of cohabitation (the number of males observed within 20 cm of the female burrow) is a good estimate of the number of male-female interactions [13], [17]. This parameter also increased linearly with male density (orthogonal linear contrast, *F*_1, 6_ = 42.9, *P* = 0. 001; Fig. 2D).

The rate of cannibalism increased linearly with male availability in excess of the higher encounter rates of males with females at higher male densities. A Poisson GLM with plot nested within treatment and controlling for the estimated per capita rate of female-male interactions (i.e. including the cohabitation rate as a covariate), showed that the interaction rate *per se* did not significantly affect the rate of sexual cannibalism (*F*_1,52_ = 0.3, *P* = 0. 615). Furthermore, there was still a significant treatment effect after the effect of cohabitation rates had been removed (orthogonal linear contrast, *F*_1,52_ = 6.5, *P* = 0.01), indicating that the higher rate of cannibalism in plots with more males did not occur solely because the encounter rate between predators (females) and prey (males) was higher.

### Field Experiment 2: Contribution of sexual cannibalism to female reproductive success

**Figure 3:**
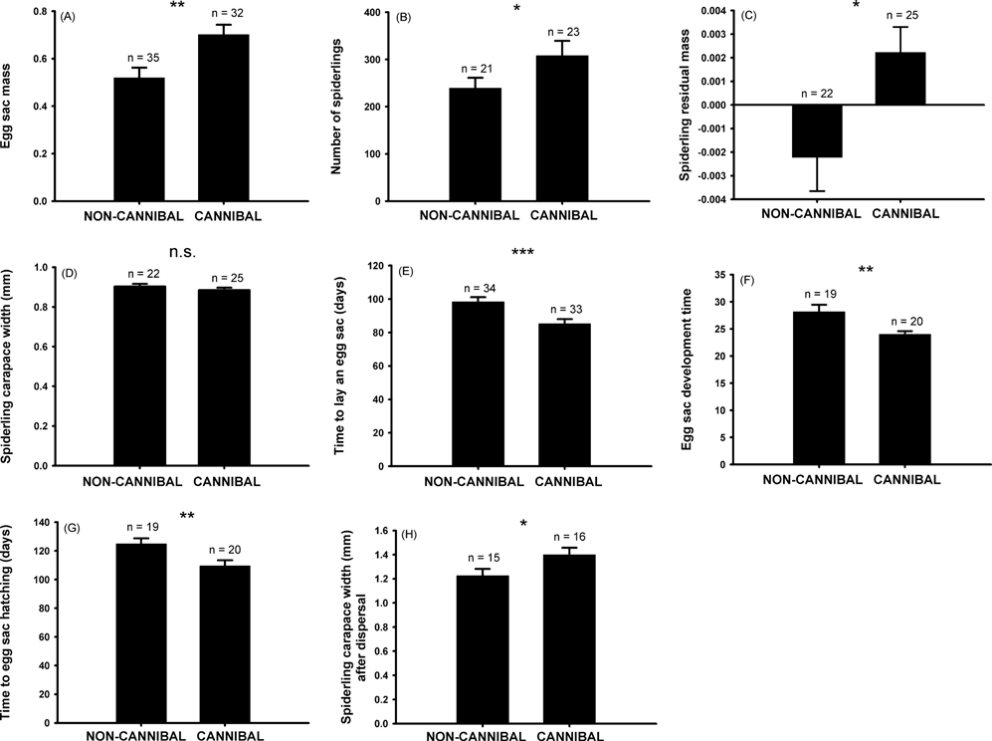
Effects of sexual cannibalism on several reproductive parameters of female *Lycosa tarantula*. Field Experiment 2 (A–G) and Field Experiment 3 (H). Solid bars and error bars represent means and standard errors, respectively, of (A) egg-sac mass; (B) number of spiderlings that hatched from the egg sac; (C) residual mass of spiderlings at hatching, calculated from a linear regression between the cubic root of weight and carapace width; (D) spiderling body size at hatching (carapace width); (E) time (days) elapsed between female maturation and production of the egg sac; (F) time (days) from production of the egg sac to hatching; (G) total development time (time from female maturation to egg-sac hatching); and (H) body size of spiderlings at the end of the dispersal period, which ranged from 1 to 3 months depending on the mother. Symbols denote the level of statistical significance: n.s., non-significant; *, *P*<0.05; **, *P*<0.01; ***, *P*<0.001.

In small field plots, single isolated females that were provided a superabundance of alternative natural prey were allowed to kill a male *L. tarantula*; half of the females (“cannibalistic”) were allowed to feed on the male, whereas the male was immediately removed from the jaws of the others, the “non-cannibalistic” females. Feeding on a cannibalized male enhanced several parameters directly related to reproductive success. Egg sacs of cannibalistic females were 40% heavier (GLM, *F*_1, 64_ = 9.5, *P* = 0.003, Fig. 3A), a difference that translated into a difference in the number and quality of offspring. Cannibalistic females had 30% more spiderlings per clutch (Poisson GLM, χ^2^_1_ = 4.8; *P* = 0.028; Fig. 3B). Egg hatching success was not significantly affected by female cannibalism (GLM, *F*_1, 42_ = 0.44; *P* = 0.50), but the spiderlings of cannibalistic females were in better condition (mass/size residuals) than the offspring of non-cannibalistic females (GLM, *F*_1, 45_ = 6.34, *P* = 0.015; Fig. 3C). However, offspring size, defined as carapace width, did not differ between treatments (GLM, *F*_1, 45_ = 1.10, *P* = 0.30, Fig. 3D).

Several parameters related to the timing of reproduction also were favoured by cannibalism. Just before laying eggs, *L. tarantula* females close the mouth of their burrow with silk. The majority (80%; 28/35) of the cannibalistic females did so, whereas only 39% (13/33) of non-cannibalistic females sealed their burrows (binomial GLM, χ^2^_1_ = 12.1; *P* = 0.0006). Cannibalistic females produced an egg sac 13 days before non-cannibalistic females (GLM, *F*_1, 65_ = 11.85, *P* = 0.001; Fig. 3E), and eggs laid by cannibalistic females developed 4 days faster than those of non-cannibalistic females (GLM, *F*_1, 37_ = 8.9, *P* = 0.005; Fig. 3F), probably due to the fact that egg sacs of non-cannibalistic females were made later in the fall, when ambient temperatures were lower. The net result of this difference in the timing of egg production was that egg sacs of cannibalistic females hatched on average 15 days earlier than those of non-cannibalistic females (GLM, *F*_1, 37_ = 7.77, *P* = 0.008; Fig. 3G).

Female survival is a major component of net reproductive rate. Mortality of females during this field experiment was high; ca. 50% died before producing an egg sac. However, sexual cannibalism did not affect female mortality (binomial GLM, *P* = 0.92), which likely was caused by heavy rainfall. Hence effects of having fed on a male were expressed solely in the reproductive parameters of egg number, spiderling body condition, timing of egg laying, and egg developmental rate.

### Field Experiment 3: Survival and growth of dispersing offspring of cannibalistic and non-cannibalistic females

Subsets of recently hatched spiderlings of each female were introduced, at two different densities, into the small field plots that had housed females in Field Experiment 2. Earlier dispersal of spiderlings from cannibalistic mothers gave them more time to grow, which translated into their having a larger body size than offspring of non-cannibalistic females at the end of this experimentally delimited dispersal period (Fig. 3H). We analyzed the developmental pattern with GLM, with female (random factor) nested within the interaction spiderling density x female cannibalism (both fixed factors). Female cannibalism had a significant effect on spiderling size (*F*_1, 54_ = 5.2, *P* = 0.029; Fig. 3H), but there were no significant effects of either density (*F*_1, 54_ = 1.0, *P* = 0.321) or the density x cannibalism interaction (*F*_1, 54_ = 0.0, *P* = 0.850). Further analysis demonstrated that cannibalism did not directly explain the larger final size of offspring of cannibalistic females. Including the dispersal date of spiderlings as a covariate in the GLM model (covariate *F*_1, 28_ = 20.5, *P* = 0.0001) resulted in no effect of the cannibalism treatment on spiderling size (GLM, *F*_1, 28_ = 0.36, *P* = 0.555). Thus, the benefit that spiderlings obtained from their mothers having experimentally fed on a male was that of more time to grow after leaving the egg sac rather than an increase in growth rate *per se*. There were significant effects of dam (mother) on final spiderling size (*F*_27, 54_ = 3.6, *P*<0.0001), suggesting genetic and/or maternal effects on growth rate.

We did not find any significant effects of female cannibalism (*F*_1, 25_ = 0.3, *P* = 0.598), density (*F*_1, 25_ = 1.2, *P* = 0.281) or their interaction (*F*_1, 25_ = 0.2, *P* = 0.663) on spiderling survival. Release date was included as a covariate (*F*_1, 25_ = 4.5, *P* = 0.044) to control for the effects of time on mortality, as longer time in the enclosures should lead to increased mortality independently of any possible treatment effects.

### Laboratory Experiment 1: Rates of cannibalism among offspring of cannibalistic and non-cannibalistic females

At the end of the dispersal period of Field Experiment 3, we randomly selected offspring of cannibalistic and non-cannibalistic females to test for possible advantages in spiderling-spiderling interactions that could lead to cannibalism. An offspring of a cannibalistic female was paired in a Petri dish with an offspring of a non-cannibalistic mother. Progeny of cannibalistic females were more likely to kill and eat the other spiderling. Cannibalism occurred in 17 out of 31 Petri dishes, with the offspring of the cannibalistic female being the cannibal ca. 75% of the time (G = 6.8; 1 d.f.; *P* = 0.009). A second statistical analysis controlling for family effects (see Methods) was also significant (Mann-Whitney U-test, Z = 2.03, *P* = 0.043). The difference in size between spiderlings (Percentage Difference in Size, PDS: [{size_large–size_small}/size_small] *100 [31]) significantly explained whether or not cannibalism would occur (binomial GLM, χ^2^_1_ = 21.6; *P*<0.0001), suggesting that the progeny of cannibalistic females had an advantage solely because they were larger and not because they benefitted in any other way (e.g. greater strength) from being the offspring of a mother who was experimentally allowed to cannibalize a male.

## Discussion

Our experiments strongly suggest that pre-copulatory sexual cannibalism occurs at a substantially high rate in natural populations of *L. tarantula*, and that this behaviour is an evolved female adaptation to a limited supply of high-quality prey. Below we evaluate the evidence supporting these conclusions and discuss several implications of our findings.

### Commonness of pre-copulatory cannibalism by *L. tarantula* females in nature

The rate of pre-copulatory sexual cannibalism by females in *L. tarantula* populations is likely quite high, as approximately a third of the females in Field Experiment 1 were observed killing and consuming a potential mate instead of copulating with him. In fact, and perhaps somewhat surprisingly, less than 5% of cannibalism by females was post-copulatory. This experiment was conducted under conditions very close to natural: female burrows were spread throughout the plots at a natural density, males were free to roam, and prey density and composition were maintained close to normal by the trapping protocol at plot boundaries. The major factor complicating the extrapolation of this cannibalism rate to a completely non-manipulated natural population is the variation in male densities produced by our intentional alteration of sex ratios in the plots, and by the high mortality of males during the experiment, probably due to bird predation, as these were the only predators that were not excluded (see Methods). In natural populations of *L. tarantula* the sex ratio varies throughout the mating season as male mortality from predation and cannibalism causes their numbers to decline more rapidly than females [13], [14]. It is also likely that the seasonal changes in sex ratio may vary from one area to another, depending upon differences between localities in predation pressure on males. However, local male availability around a female burrow is not only influenced by the population sex ratio, but also by the behaviour of males, who show great variation in moving rates in search of females (data collected during this study and to be published elsewhere). The female should respond to variation in local male availability, since this may be the only estimate of male availability that she can assess.

Comparison with other studies of *L. tarantula* supports the conclusion that the range in local male availability across our treatments was within the natural range. In Field Experiment 1 the number of different males observed encountering each female ranged from 1.0 to 3.6 across the male-density treatments, which we conclude likely falls within the natural range for two reasons. First, in a previous study of this population in Almería, employing a comparable sampling effort, we found that at the end of the mating season, when males are a scarce resource [13], the number of males observed visiting each female was 1.3±0.1—a number equal to that observed in the Medium male-availability treatment in the current study (1.3±0.4). Secondly, data from another population [14], in which sampling effort was ¼ that of the present study (females were only visited once every other night during the mating season), showed that across the entire season each female was visited by at least 1.9±1.4 males. Conservatively assuming that we had missed half of the visitations in that study leads to an estimated visitation rate of ∼3.8 different males visiting each female on average, which is very close to the visitation rate observed in the High male-availability treatment in the present study (3.5±0.5). Thus, our male-density manipulations yielded encounter rates that are within the range found in completely natural, non-manipulated populations.

### Evidence that pre-copulatory cannibalism in *L. tarantula* is adaptive

How could high rates of pre-copulatory cannibalism be adaptive–how can natural selection favour the killing of a mate by a female before she has gotten his sperm? Arnqvist and Henriksson (1997) proposed that under conditions in which the adult female is not food-limited, the “aggressive spillover” hypothesis could explain the evolution of pre-mating sexual cannibalism, which would be the consequence of aggressive predatory behaviour that is adaptive at an earlier life stage and that continues to be expressed in the mature female, even though it does not increase female fitness (in comparison to females who could suppress the expression of this genetically determined behaviour) [6]. Four facts argue strongly against the spillover hypothesis as the major explanation for the evolution of pre-copulatory sexual cannibalism in *L. tarantula*: (1) male *L. tarantula* are high-quality prey whose consumption improves several parameters of reproductive output (this study); (2) negative effects of strong food limitation experienced by juvenile female *L. tarantula* appear to be offset by cannibalizing mature males [14]; (3) in the current study most *L. tarantula* females who cannibalized males had already mated, and hence already had received sperm (although the possibility of a fitness cost remains if additional matings increase fecundity or improve fitness by increasing genetic diversity of the offspring); only 8% of females in Field Experiment 1 and 9% in Field Experiment 2 killed a male as a virgin; (4) females are more likely to attack males if their availability is higher (current study); a greater availability of males increases the probability that a female who has cannibalized a potential mate will encounter another one before she lays eggs. Taken together, the above findings strongly support the adaptive foraging hypothesis [3] as an explanation for the evolution of pre-copulatory sexual cannibalism by female *L. tarantula*.

However, in our results some element of “aggressive spillover” cannot be ruled out completely, because by killing males, some female *L. tarantula* risk remaining unmated. In Field Experiment 1, three out of the six virgin females who killed an approaching male remained unmated because they were in the Low male-availability treatment. This treatment may mimic what happens at the very end of the mating season, when males are scarce and some females remain unmated [13]. Early-maturing females, who have more time to find a mate and are likely food-limited, should gain more by killing the first approaching male. However, in the current study there was no relationship between the tendency to cannibalize as a virgin and the timing of maturation, indicating that virgin females who attacked males were not early-maturing females. Therefore, some of the tendency towards sexual cannibalism by virgins may be misdirected aggression, spillover from (1) aggressive behaviour related to foraging for prey, which occurs at night when males are near the burrow mouth [17]; and (2) cannibalistic behaviour directed at other mature females, which is a component of territorial defense [25]. Evidence suggests strongly, however, that any spillover effect, if present, is a minor component of *L. tarantula* sexual cannibalism, which appears to be primarily an adaptive foraging strategy.

In *L. tarantula* pre-copulatory cannibalism appears to be adaptive behaviour that has evolved to overcome negative effects of food limitation on female reproductive rate. Females that had cannibalized a single male produced more offspring with a better body condition. These females also laid their egg sacs sooner in the season, which promoted more rapid development because of warmer temperatures. A similar pattern was found in the wolf spider *Pardosa milvina* in which females fed high-quality diets in the laboratory produced eggs sacs more rapidly than females reared on a low-quality diet [22]. The result is that earlier dispersal of the spiderlings of cannibalistic females allowed them to grow to a larger size by the end of the season. These effects of cannibalism were directly due to an alleviation of food limitation, and not due to any possible genetic correlation between cannibalistic behavior and aggressive foraging behavior for prey, because in Field Experiment 2 females in both treatments had killed a male. By permitting only half of these females, selected at random, to feed completely on the male that we offered to them, we ruled out any possibility that the benefit of a male as a meal to the cannibalistic female was a spurious artefact of the fact that females who tend to be more cannibalistic may be more aggressive and thereby more successful in capturing other prey [2], [6]. This benefit of cannibalism to the female is consistent with the fact that males are several times larger than alternative prey [25], which makes a single male a more valuable prey simply in terms of calorie content.

The male appears to be a valuable source of prey not only because it is a larger bundle of calories than the alternative prey available to *L. tarantula*. In Field Experiment 2 the females had available to them a superabundance of prey species found in nature, yet consuming a single male dramatically improved reproductive output. Since calories are substitutable for a generalist predator (all other things being equal, such as prey defensive behaviors, digestibility of the prey tissues, etc.), it must not only be the calories contained within males that are important for female *L. tarantula*. Other evidence supports this conclusion. For example, cannibalistic females were more likely to close their burrows with silk. The quality of the silk produced by spiders closely depends on the quality of the prey in the diet [32], suggesting that females perhaps alleviate nitrogen limitation by feeding on males. Nitrogen limitation of female *L. tarantula* in a different population is suggested by indirect measurements of nutrient content in females that had been collected from the field as ante-penultimate instars [33]. Additional evidence for male *L. tarantula* being high-quality prey comes from the fact that most alternative prey for *L. tarantula* females are detritivores [25], which in aquatic food webs have higher C∶N ratios than arthropod predators [34], [35]. Thus, sexual cannibalism in *L. tarantula*, in addition to providing a large packet of easily extractable calories, may secure the additional benefit of redressing nutritional imbalance experienced by females, which seems particularly crucial during the egg-ripening stage [33]. It would be worthwhile to know whether or not female *L. tarantula*, in addition to attacking males at rates that reflect the extent to which they have experienced a scarcity of natural prey [14], are more likely to attack males if they are suffering a nutritional imbalance, as it has been shown for arthropod predators exposed to heterospecific prey [24]. Nutritionally unbalanced wolf spiders have been shown to be less efficient at feeding on prey other than conspecifics [36]. This phenomenon may explain why, in our experiment, the females that had not fed on a male laid smaller egg sacs despite having large amounts of alternative prey in the enclosures (see Methods). Therefore, redressing nutritional imbalance by feeding on a male may improve how well females process other prey. Experiments with wolf spiders suggest that food limitation may lead to enzyme limitation that in turn constrains foraging efficiency [37].

Female flexibility in cannibalistic tendencies suggests that pre-copulatory cannibalism by *L. tarantula* is primarily an adaptive foraging behaviour, in which females weigh the benefits of males as sperm donors or prey. Despite the fact a male is an excellent food resource, females generally behave adaptively towards approaching males. Several results support this interpretation of flexible female behaviour towards males. First, few virgin females (8% in Field Experiment 1, 9% in Field Experiment 2) killed approaching males. The proportion of females that killed approaching males rose to 25% once females had secured sperm for egg production (Field Experiment 1), a pattern found in other studies [9], [38]–[40]. Second, females tend to kill males at a higher rate as more males are available (Field Experiment 1). This difference was statistically significant even after controlling for the female *per capita* encounter rate with males. Thus, females were not preying on males at a higher rate merely because the encounter rate between females (predators) and males (prey) was higher in plots with higher male availability. This result strongly suggests that a female's decision to kill a potential mate was based at least partly on her assessment of male availability. A laboratory experiment with the fishing spider *Dolomedes triton* [41], in which females were experimentally induced to assess the environment as rich or poor in males, found similar results. However, Johnson's laboratory experiment [41] is not easily interpretable, as its design did not control whether females assessed males as such, or whether females were mistaking males as highly mobile prey. Although Johnson used juvenile *D. triton* presumably to control for the possibility that females were mistaking males for potential prey, the mobility of juvenile *D. triton* is much less than that of females [42], which in turn, as in most spiders, must be lower than that of males [28], [43]. Our field manipulations of actual male availability eliminated alternative explanations for the observed pattern, thereby providing strong support for the hypothesis that females behave adaptively towards approaching males, being more likely to attack once they have assessed the environment to have a high availability of males.

### Fitness benefits to the cannibalistic female via progeny traits

Surprisingly, the better body condition of the offspring of cannibalistic females did not translate into any measurable fitness benefits after spiderling dispersal (Field Experiment 3). This apparent paradox may be explained by the fact that in nature not all spiderlings disperse at once, as we experimentally induced. In fact, for this species there appear to be two dispersal peaks of siblings: one before and another after the winter [44]–[46]. Perhaps spiderlings in better condition have less of an immediate need for food and therefore tend to remain with their mothers during winter. Indeed, the relative benefits of a better body condition may depend upon the net fitness benefit of dispersing before or after the winter, which may change from year to year depending on variation in weather conditions [44], [46].

### The male perspective

Sexual cannibalism occurs very rarely after mating in *L. tarantula*, as we observed only one case of post-mating sexual cannibalism. Therefore, most cannibalized males are eaten by females with whom they have not mated, which means that the selective benefit of sexual cannibalism to most males is zero. Hence, female cannibalistic behaviour should impose a strong selective pressure on males to evolve counter-adaptations to avoid female attacks. Previous research on *L. tarantula* suggests that males do not approach females randomly, but instead, approach females at times of day when predation risk is lowest [17] and/or they preferentially approach females that present a lower predation risk [13]. Females, on the other hand, apparently have been selected to behave adaptively towards males. It remains to be investigated whether this female adaptive behaviour (i.e., attacking males only after ensuring sperm) has evolved from selection imposed by males that preferentially approach non-aggressive females or because high male mortality during the mating season [28], [43], [47] entails a high risk of remaining unmated for females in natural populations.

### Conclusions

The results of our research are particularly relevant to understanding the ecology and evolution of sexual cannibalism because (1) the data were gathered in field experiments conducted under natural conditions; (2) sexual cannibalism [1] and cannibalism in general [15] are widespread among spiders; and (3) spiders are abundant and ubiquitous in terrestrial systems [48]. Data on sexual cannibalism were obtained under natural rates of encounter between males and females, with the same opportunities for males to escape female attacks as occur in nature. The experiments revealed a high incidence of sexual cannibalism in a natural population of *L. tarantula*, and demonstrated that a diet including a single male is much better than a diet consisting only of alternative natural prey, strongly suggesting that sexual cannibalism may help females to alleviate calorie and nutrient limitation. Thus, in *L. tarantula* pre-copulatory sexual cannibalism is an evolved, adaptive foraging strategy.

## Materials and Methods

### Species and study site

The Mediterranean tarantula (*Lycosa tarantula*) is a burrowing wolf spider with a 2-year life cycle [49], [50]. Juvenile spiders wander until they are one-year old, at which time they settle in burrows [51]. Maturation occurs at 21–22 months of age and the mating season takes place between June and August. All field experiments were conducted outside the border of the Cabo de Gata-Níjar Natural Park, Almería, in southeastern Spain, on the same study site in which some of the previous studies with this species were performed [13], [17], [25].

### Field Experiment 1: Natural rates of pre-copulatory sexual cannibalism and the influence of male availability on cannibalism rate

#### Experimental design

Sub-adult spiders of both sexes were collected from nearby areas from 10 to 25 May 2005. Virgin females (n = 72) were added to nine 12×12-m plots that were set in a 3×3 array of 50×50 m and from which all *L. tarantula* had been removed. Eight females were added to each plot by introducing them into artificial burrows [25] equidistantly spaced within the plot. With this procedure we assured that all plots had identical female densities with the same spatial dispersion pattern. Around the entrance of natural burrows the spider constructs a turret, which has been shown to improve spider survival [52]. The turret was removed from the original burrow from which the captured spider had been removed, and was placed around the mouth of the artificial burrow. These burrows, which were constructed entirely of materials that spiders use to make natural burrows, have been found to promote natural burrowing behaviors, as all introduced spiders remain in them, commence excavating them further to meet their own requirements, and immediately fix the turret with silk after being introduced into the burrow by us. A previous experiment [25] with these burrows that included a control treatment for the effect of the burrow itself showed that the artificial burrow had no effect on spider mortality. The burrow density utilized in this experiment was similar to previous estimates of female density in this population [50]. Each plot was randomly assigned to one of three male-availability treatments: Low (8 males), Medium (16 males) and High (32 males). The original goal was to establish a 1∶1 sex ratio for the Medium treatment, with 4 and 16 males in the Low and High treatments, respectively. However, unexpected high mortality of males soon after the additions, probably from bird predation, forced us to add additional males to all treatments in order to achieve natural rates of encounter between males and females. With this modification we established rates of encounter between males and females in the Medium treatment that were very close to those observed in nature (see Discussion). From 25 May to 17 July we added 65 males of known virginity (collected as subadults from the surrounding area, placed in artificial burrows outside the study plots, and daily checked for maturation) and 70 males of unknown mating history collected as adults from the surrounding area (18–19 males per week for 7 weeks). Males from both groups were randomly assigned to experimental treatments with the restriction that the ratio of virgin males to males of unknown mating history was the same for each plot. Males were released in the plots at night in order to prevent excessive exposure to heat. We placed 5 bricks in each plot (one in the centre and one in each corner) as shelter from excessive heat for recently introduced males.

A 30-cm wide continuous trench (15 cm in depth) along the perimeter of each plot housed two contiguous pitfall traps made of sheet metal, which made it difficult for trapped arthropods to escape. The trench traps were emptied at dawn and dusk, and also during the day when conditions made it likely that captured animals would become overheated if left in the traps all day. The continuous monitoring of two pitfall traps allowed us to document and control the natural flow of walking migrating prey in and out of the plots. All arthropods >0.5 mm (and thus potential prey of *L. tarantula*) that were captured in the outer pitfall trap were introduced into the plots, and prey found in the inner trap were released outside the plot. Since scorpions are known to be important predators of female *L. tarantula* [13], [52], we did not introduce into the plots scorpions that were trapped in the outer trench traps. At the beginning of the experiment a single scorpion killed and consumed two females, which convinced us that it would be too risky for the experiment to allow scorpions to immigrate into the plots. All *L. tarantula* males found in the inner trap were returned to the centre of the plot. Predation by foxes (*Vulpes vulpes*), a major source of mortality for *L. tarantula* [25], [26], was prevented by installing a 50-cm electric fence around the 0.25 ha area containing the nine plots. However, bird predation, which was presumably very high on males as judged by the relatively low survival times (mode = 5 days), was not prevented by this design.

#### Marking and monitoring

Before being placed in the plot, each spider was weighed (nearest 0.01 g) and its carapace (CW) and abdomen (AW) widths were measured (nearest 0.01 mm). Each spider was uniquely marked–females with markings on the legs [14], [50], males with either leg markings and bee tags (of which we used two colors, orange and green), or only with leg markings. An evaluation of male survival (defined as days remaining in the plots) using survival regression analysis [31] revealed that the three different markings (orange or green bee tags or marking on the legs only) did not differentially affect male mortality (Accelerated Failure Time Survival Model with Weibull distribution, χ^2^_2_ = 2.8; *P* = 0.246). Since release time had a significant effect on mortality, with males that were released later in the season surviving for a shorter time, we included release time as a covariate in the model (χ^2^_1_ = 3.9; *P* = 0.049).

#### Behavioural data

Throughout the season each female burrow was closely monitored for cannibalistic and mating behaviours. A male remains near the entrance of a female's burrow up to several days (“cohabitation”), moving slightly farther from the entrance at night, when the female comes out to hunt prey [17]. Mating occurs inside the burrow, but only during daylight hours [13], [14], [17], [53]. Because of this known activity pattern, we expected to detect pre-copula cannibalisms mainly at night. Preliminary observations suggested that the handling time of cannibalized males is several hours. Thus, in order to maximize the chances of observing cannibalism [13], every female was visited once each night and again early in the morning (see below). Mating and cohabitation were monitored during the day by visiting females at 1-h intervals from 8 am to 3 pm, the time window when most copulations occur in nature [13]. This observation schedule provided a nearly complete picture of the sexual and cannibalistic activities of the spiders in the plots.

#### Statistical analyses

Most tests were performed using the General Linear Model (GLM) applied to plot means. Since we predicted that mating and cannibalism rates (“rate” defined as the mean number of occurrences per plot or per treatment) would increase linearly with male availability, we used orthogonal linear contrasts for testing planned comparisons. We first ran all analyses including the spatial coordinates of the plots (X,Y) in order to determine if a spatial autocorrelation might contribute to patterns that were otherwise being ascribed solely to treatment effects. Because none of the coordinates was significant in any of the tests (all *P*>0.3), and the results remained qualitatively the same when they were removed, we removed the spatial coordinates from the statistical models presented here. For hypotheses in which female covariates needed to be included in the model, we nested plot within treatment in order to control for plot effects. We included plot as a fixed factor because it has been shown that when the number of treatment levels is below 10 and the potentially random effect does not absorb the fixed effect, the inclusion of random factors results in highly unreliable estimates [54], [55]. Since the dependent variable in the model was discrete (e.g. number of cannibalistic events, number of matings), we used Generalized Linear Models with Poisson distributions controlled for overdispersion [56]. We used STATISTICA 8.0 except for orthogonal contrasts in Poisson GLM, for which we used SAS 9.0.

### Field Experiment 2: Contribution of sexual cannibalism to female reproductive success

#### Experimental design

This experiment was conducted in the following year (May–November 2006). To ensure the virginity of mature females that were used in the experiment, sub-adult females (n = 80) were placed in artificial burrows (see above) and isolated in field enclosures 1 m×1 m×30-cm high, an area that approximates the smallest territory size of a female in this population [25], [29]. The enclosures were then covered with 0.5-cm mesh screening to prevent bird predation. This design allowed us to control the availability of natural prey species, and to manipulate the nutritional benefits of cannibalism by removing a recently killed male from the jaws of half of all females that had killed a male.

#### Induced mating

An adult male collected from the field (i.e., of unknown mating history) was introduced to the enclosure of every female one week after she had moulted to maturity. We removed the male and introduced a new male daily until the first complete mating occurred. The aim was to ensure that all females mated, so that it would be possible to test if the cannibalism treatment (described below) altered female reproductive success. Males, which had been randomly assigned to each of the 80 enclosures containing the female burrows, were released daily into a 20×20×10-cm metal cage placed on top of the burrow entrance; 8 female mating trials were run per day. This male enclosure ensured normal male courtship, as normal cohabitation distances during the night, the time prior to mating, are 15–20 cm [17]. On the first introduction of a male, some females cannibalized their potential partner instead of copulating with him. All males found dead in the female's jaws were removed and weighed. Statistical models were run including this extra-male intake as a covariate, but because this variable was never significant (*P*'s>0.2), we excluded it from the final analyses. The average mass extracted by females in these “secondary” cannibalistic attacks was 0.12±0.03 g, which is ca. one-third the mass of a single natural prey item (see below). Females that killed the male were offered a different male the next day and so on until a complete mating was achieved. Males were never used more than once.

This experiment was part of a larger “polyandry” experiment in which half of the females were offered 4 additional males as potential mates, but were not allowed to cannibalize them. The effect of this additional treatment and its interaction with the cannibalism treatment were not significant for any of the variables measured here (*P*'s>0.16). These results will be published separately as part of another study on sexual conflict.

#### Cannibalism treatment

One week after each female had first mated she was presented with a male that had been kept in a freezer for 10 minutes to make him lethargic and susceptible to female attack. Females were then randomly assigned to one of two cannibalism treatments: Cannibalistic or non-cannibalistic. In the non-cannibalistic treatment, the male was removed from the female's jaws 10 minutes after being killed, thus preventing the female from cannibalizing him. Females in the non-cannibalistic treatment extracted on average 7.2±0.4% of the male mass during this 10-minute period. In contrast, in the cannibalistic treatment females were allowed to completely consume the males they had killed and presumably consumed most of the male. Assessing how much remained after allowing the experimentally cannibalistic females to consume the male was not feasible because only a portion of the remains is left outside the burrow; the rest is deposited inside the burrow, making full recovery unlikely. Once a week females in both treatments were given 10 of the most frequently consumed natural prey items: darkling beetles (Tenebrionidae) and woodlice (Isopoda) [25] in a proportion that depended on natural availability and which changed from week to week. Since the natural rate of feeding is lower than one prey item per night [50], this rate of prey supplementation should have mimicked an unlimited food supply. Female feeding behaviour corroborates the prediction that this rate of prey supply provided an excess of prey. We counted and weighed the prey that remained alive in the enclosures of 7 experimentally non-cannibalistic females that had laid an egg sac. The average number of live prey in each enclosure was 35±2.8 (SE). The average weight was 0.31±0.03 g, which translates into an average total biomass per enclosure of 10.6±0.9 g, which is about 7 times the mass of a single adult male *L. tarantula* (1.47±0.02 g; N = 139).

#### Fecundity estimates

After mated females have acquired enough food resources for egg development, they seal the mouth of their burrow with silk, probably as a defence against predators [50]. Females at this stage were removed from their burrows and isolated in extractable PVC burrows (20 cm×2.5-cm diameter) until their spiderlings had emerged from the egg sac. The mouth of these burrows, which were located outside the experimental plots, was covered with 0.5 mm-mesh netting that allowed us to monitor egg laying and spiderling hatching without disturbing the female. Hatchlings were counted and a randomly selected sub-sample (n = 10 per female) was weighed and their carapace widths (CW) measured [26]. The remaining offspring were either released back into the field or used in another experiment testing for offspring performance (see below). Females inside burrows suffered high mortality between the stages of egg-sac development and egg hatching, probably from high amounts of rainfall. Thus we were able to assess estimates of reproductive fitness for only 56% of the females (n = 45). The fitness estimates were egg sac mass, number of spiderlings, spiderling size (CW), spiderling body condition (the residuals from the linear regression of the cubic root of mass on carapace width), hatching success (whether or not the spiderlings were able to break out of the egg sac), and hatching rate (the ratio of the number of spiderlings hatched to the total number of eggs laid).

#### Statistical analyses

We used GLM to test for differences between treatment groups. For binary response variables we used GLM with a binomial error distribution, and for discrete response variables (counts) we used GLM with a Poisson distribution controlled for overdispersion [56]. Since body size did not affect either the mass of the egg sac nor the number of offspring, we did not include it as a covariate for our final analyses. STATISTICA 8.0 was used for all analyses.

### Field Experiment 3: Survival and growth of dispersing offspring of cannibalistic and non-cannibalistic females

As a means to evaluate effects of the cannibalism treatment on offspring performance, we used hatched spiderlings from the above experiment. A sub-sample of full-sib spiderlings from 31 females in the cannibalistic and non-cannibalistic treatments (16 and 15 females, respectively) was returned to the 1×1-m field enclosures used to house the females before they laid an egg sac. Low fecundity in some females and the need to allocate 10 spiderlings for body measurements (see above) prevented us from utilizing offspring from all the females that survived and laid egg sacs. In order to prevent spiderlings from aggregating in the old female burrow, we filled it with stones and dirt. We introduced spiderlings at two densities (high, 42/m^2^; and low, 21/m^2^). This range in density of dispersing offspring was based upon previously collected data on the density and fecundity of females in this population of *L. tarantula* [50], and also data from the current study. The spiderling-density treatment was crossed with the cannibalism treatment that their mothers experienced (cannibalistic-high, n = 9; cannibalistic-low, n = 7; non-cannibalistic-high, n = 9; non-cannibalistic-low, n = 6). Due to variation in the timing of spiderling dispersal from the mother, the 1 m×1 m-plots with dispersed spiderlings were set up from 19 August through 10 November 2006; the experiment ended 6 December 2006. At the end of the experiment, all surviving spiderlings were removed, weighed and measured (CW and AW). In order to uncover family differences in growth rates (either from maternal effects distinct from having fed experimentally on a male, or from genetic differences), we nested dam (mother) as random factor within the cannibalistic treatment in a GLM. STATISTICA 8.0 was used for all analyses.

### Laboratory Experiment 1: Rates of cannibalism among offspring of cannibalistic and non-cannibalistic females

Field and laboratory microcosm experiments have demonstrated that spiderling-spiderling cannibalism can be a significant factor regulating densities of wolf spiders [57], [58]. In the laboratory we tested whether or not the larger size attained by the offspring of cannibalistic females, due to their earlier hatching and dispersal, gave them an advantage over the offspring of non-cannibalistic females in spiderling-spiderling encounters. We used spiders that had survived to the end of Field Experiment 3. One offspring of a cannibalistic female was paired with an offspring of a non-cannibalistic female in 9-cm Ø Petri dishes (*n* = 31) that contained a 2-mm deep substrate of fine soil from the study site. We started the experiment in the afternoon and checked for cannibalism every hour for 12 hours. The data analysis was complicated by the need to include more than one offspring from the same mother. We first present the results of a G-test done on the data ignoring genetic relatedness. Then, in order to remove possible problems of interpretation due to pseudoreplication, we employed a randomization procedure that consisted of selecting at random one individual from each Petri dish and averaging the response (cannibalistic “1” or non-cannibalistic “0”) across mothers, using each mother as a replicate, for analysis with the Mann-Whitney U-test. This non-parametric test was used because averages constructed from 1's and 0's are highly skewed. STATISTICA 8.0 was used for all analyses.
